# Modulation Spectra Morphological Parameters: A New Method to Assess Voice Pathologies according to the GRBAS Scale

**DOI:** 10.1155/2015/259239

**Published:** 2015-10-18

**Authors:** Laureano Moro-Velázquez, Jorge Andrés Gómez-García, Juan Ignacio Godino-Llorente, Gustavo Andrade-Miranda

**Affiliations:** ETSIST, Universidad Politécnica de Madrid, Campus Sur, Carretera de Valencia km 7, 28031 Madrid, Spain

## Abstract

Disordered voices are frequently assessed by speech pathologists using perceptual evaluations. This might lead to problems caused by the subjective nature of the process and due to the influence of external factors which compromise the quality of the assessment. In order to increase the reliability of the evaluations, the design of automatic evaluation systems is desirable. With that in mind, this paper presents an automatic system which assesses the Grade and Roughness level of the speech according to the GRBAS perceptual scale. Two parameterization methods are used: one based on the classic Mel-Frequency Cepstral Coefficients, which has already been used successfully in previous works, and other derived from modulation spectra. For the latter, a new group of parameters has been proposed, named *Modulation Spectra Morphological Parameters*: MSC, DRB, LMR, MSH, MSW, CIL, PALA, and RALA. In methodology, PCA and LDA are employed to reduce the dimensionality of feature space, and GMM classifiers to evaluate the ability of the proposed features on distinguishing the different levels. Efficiencies of 81.6% and 84.7% are obtained for Grade and Roughness, respectively, using modulation spectra parameters, while MFCCs performed 80.5% and 77.7%. The obtained results suggest the usefulness of the proposed *Modulation Spectra Morphological Parameters* for automatic evaluation of Grade and Roughness in the speech.

## 1. Introduction

With the aim of diagnosing and evaluating the presence of a voice disorder clinicians and specialists have developed different assessment procedures [1] such as exploration using laryngoscopic techniques, acoustic analysis, or perceptual evaluations. The latter is widely used by clinicians to quantify the extent of a dysphony. Some well-known perceptual evaluation procedures are the Buffalo Rating Voice Profile [2], Consensus Auditory Perceptual Evaluation of Voice (CAPE-V) [3], and GRBAS [4]. The main problem with perceptual analysis is the high intra/interrater variability [5, 6] due to the subjectivity of the assessment in which the experience of the evaluator, his/her physical fatigue, mental condition, and some other factors are involved. Hence, means such as acoustic analysis based on signal processing might be valuable in clinical scenarios, providing objective tools and indices which can directly represent the level of affection or at least help clinicians to make a more reliable and less subjective perceptual assessment. This noninvasive technique can complement and even replace other invasive methods of evaluation.

Besides, the large amount of improvements in the field of speech signal processing is addressed mostly to areas such as speech or speaker recognition. Many of these advances are being transferred to biomedical applications for clinical purposes; some recent examples are related to different uses such as telemonitoring of patients [7], telerehabilitation [8], or clinical-support systems [9]. However, there is a substantial quantity of research to be done for further enhancements. Roughly speaking, most of the studies in this field can be divided into three main categories: the first one is focused on developing automatic detectors of pathological voices [10–14] capable of categorizing voices between normal and pathological; the second group works with classifiers of pathologies [11, 15, 16] which consists in determining the speech disorder of the speaker using the acoustic material; and the third and last group aims to evaluate and assess the voice quality [8, 17–23]. The present study can be framed in the third mentioned category, highlighting the fact that the main goal is the development of new parameterization methods.

The essential common characteristic of all the automatic systems found in the literature is the need to extract a set of parameters from the acoustic signal to accomplish a further classification task. Regarding these parameters, some works use* amplitude perturbations* such as Shimmer or Amplitude Tremor Intensity Index (ATRI) [24–26] as input features while others are centered on* frequency perturbations* using Jitter [8, 25, 26], frequency and cepstral-based analysis [8, 13, 14, 16, 17, 27, 28], *F*_0_ Tremor Intensity Index (*F*_0_TRI) [24, 26], or Linear Predictive Coding (LPC) [29].* Noise-based parameters* [30, 31] and* nonlinear analysis features* [12, 18, 25, 32] are likewise widely used in this kind of automatic detectors. Moreover, other varieties of feature-extraction techniques such as biomechanical attributes or signatures can be applied for the same purposes [10].

Focusing on the third kind of the aforementioned categories of detectors, those assessing the quality of voice, some are employed for simulating a perceptual evaluation such as GRBAS. For instance, several classification methods were used in [19, 20] to study the influence of the voice signal bandwidth in perceptual ratings and automatic evaluation of GRBAS Grade (*G*) trait using cepstral parameters (Linear Frequency Spectrum Coefficients and Mel-Frequency Spectrum Coefficients). Efficiencies up to 80% were obtained using Gaussian Mixture Models (GMM) classifiers and leave-x-out [33] cross-validation techniques. Similar parameterization methods were used in [9] to automatically evaluate *G* with a Back-and-Forth Methodology in which there is feedback between the human experts that rated the database and the automatic detector, and vice versa. On [22] a group of 92 features comprising different types of measurements such as noise, cepstral and frequency parameters among others were used to detect GRBAS Breathiness (*B*). After a reduction to a four-dimensional space, a 77% of efficiency was achieved using a 10-fold cross-validation scheme. Authors in [34] fulfilled a statistical study of acoustic measures provided by two commonly used analysis systems,* Multidimensional Voice Analysis Program* by Kay Elemetrics and* Praat* [35] obtaining good correlations for *G* and *B* traits. On [21] Mel-Frequency Spectrum Coefficients (MFCCs) were utilized obtaining 68% and 63% of efficiency for Grade and Roughness (*R*) traits, respectively, using Learning Vector Quantization (LVQ) methods for the pattern recognition stage but without any type of cross-validation techniques. The review of the state of the art reports that only [36] has used the same database and perceptual assessment used in the present study. The mentioned work proposed a set of complexity measurements and GMM to emulate a perceptual evaluation of all GRBAS traits, but its performance does not surpass 56% for *G* or *R*.

In general, results seldom exceed 75% of efficiency; hence, there is still room for enhancement in the field of voice quality automatic evaluation. Thus, new parameterization approaches are needed and the use of Modulation spectrum (MS) emerges as a promising technique. MS provides a visual representation of sound energy spread in acoustic and modulation axes [37, 38] supplying information about perturbations related to amplitude and frequency modulation of the voice signal. Numerous acoustic applications use these spectra to extract features from audio signals from which some examples can be found in [39–42]. Although there are few publications centered in the characterization of dysphonic voices using this technique [11, 12, 23, 43], it can be stated that MS has not been studied deeply in the field of the detection of voice disorders and specially as a source of information to determine patient's degree of pathology. Some of the referred works have used MS to simulate an automatic perceptual analysis but, to the best of our knowledge, none of them offer well-defined parameters with a clear physical interpretation but transformations of MS which are not easily interpretable, limiting their application in the clinical practice.

The purpose of this work is to provide new parameters obtained from MS in a more reasoned manner, making them more comprehensible. The use of this spectrum and associated parameters as support indices is expected to be useful in medical applications since they provide easy-to-understand information compared to others such as MFCC or complexity parameters, for instance. The new parameterization proposed in this work has been used as the input to a classification system that emulates a perceptual assessment of voice following the GRBAS scale in *G* and *R* traits. These two traits have been selected over the other three (Aesthenia (*A*), Breathiness, and Strain (*S*)) since its assessment seems to be more reliable. De Bodt et al. [5] point that *G* is the less unambiguously interpreted and *R* has an intermediate reliability on its interpretation. These conclusions are coherent with those exposed in [44, 45]. Similar findings are revealed in [6] which considers *R* as one of the most reliable traits when using sustained vowel /*ah*  :  / as source of evaluation. It is convenient to specify that each feature of the GRBAS scale ranges from 0 to 3, where 0 indicates no affection, 1 slightly affected, 2 moderately affected, and 3 severely affected voice regarding the corresponding trait. Thus evaluating according to this perceptual scale means developing different 4-class classifiers, one for each trait.

In this work, the results obtained with the proposed MS-based parameters are compared with a classic parameterization used to characterize voice in a wide range of applications: Mel-Frequency Cepstral Coefficients [46]. MFCCs have been traditionally used for speech and speaker recognition purposes since the last two decades and many works use these coefficients to detect voice pathologies with a good outcome.

The paper is organized as follows: Section 2 develops the theoretical background of modulation spectra features.  Section 3 introduces the experimental setup and describes the database used in this study.  Section 4 presents the obtained results. Lastly, Section 5 presents the discussions, conclusions, and future work.

## 2. Theoretical Background

### 2.1. Modulation Spectra

This study proposes a new set of parameters based on MS to characterize the voice signal. MS provides information about the energy at modulation frequencies that can be found in the carriers of a signal. It is a three-dimensional representation where abscissa usually represents modulation frequency, ordinate axis depicts acoustic frequency, and applicate, acoustic energy. This kind of representation allows observing different voice features simultaneously such as the harmonic nature of the signal and the modulations present at fundamental frequency and its harmonics. For instance, the presence of tremor, understood as low frequency perturbations of the fundamental frequency, can be easily noticeable since it implies a modulation of pitch as an usual effect of laryngeal muscles improper activity. Other modulations associated with fundamental or harmonic frequencies could indicate the presence of a dysfunction of the phonatory system. Some examples can be found in [11].

To obtain MS, the signal is filtered using a short-time Fourier transform (sTFT) filter bank whose output is used to detect amplitude and envelope. This outcome is finally analyzed using FFT [47] producing a matrix *E* where MS values at any point can be represented as *E*(*f*_*a*_, *f*_*m*_). The columns at *E* (fixed *f*_*m*_) are modulation frequency bands, and rows (fixed *f*_*a*_) are acoustic frequency bands. Therefore, *a* can be interpreted as the index of acoustic bands and *m*, the index of modulation bands while *f*_*a*_ and *f*_*m*_ are the central frequencies of the respective bands. Due to the fact that values *E*(*f*_*a*_, *f*_*m*_) have real and imaginary parts, the original matrix can be represented using the modulus |*E*| and the phase arg(*E*) of the spectrum. Throughout this work, the MS has been calculated using the Modulation Toolbox library version 2.1 [48]. Some different configurations can be used to obtain *E*, where the most significant degrees of freedom are the use of coherent or noncoherent (Hilbert envelope) [49] modulation, the number of acoustic bands, and acoustic and modulation frequency ranges. The three-dimensional phase unwrapping techniques detailed in [50] are used to solve the phase ambiguity problems which appear when calculating arg(*E*(*f*_*a*_, *f*_*m*_)).

**Figure 1:**
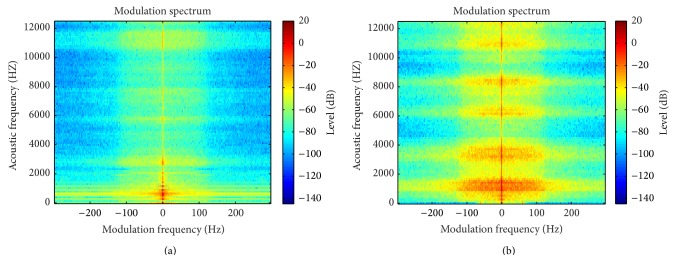
MS modulus of a normal voice (a) and pathological voice of a patient with gastric reflux, edema of larynx, and hyperfunction (b).

Figure 1 shows an example of MS extracted from two different voices on which the voice of a patient with gastric reflux, edema of larynx, and hyperfunction exhibits a more spread modulation energy in comparison to a normal voice.

However, one of the principal drawbacks of MS is that it provides a large amount of information that can not be easily processed automatically due to limitations of the existing pattern recognition techniques and voice disorders databases available. In this sense, MS matrices have to be processed to obtain a more compact but precise enough representation of the represented speech segments. Thus, after obtaining the MS, some representative parameters are extracted to feed a further classification stage. With this in mind, a new group of* Morphological Parameters* based on MS is proposed in this work: centroids [51] (MSC), dynamic range per band (DRB), Low Modulation Ratio (LMR), Dispersion Parameters (CIL, PALA, and RALA), Contrast (MSW), and Homogeneity (MSH). All these parameters use the MS modulus as input source, except the last two which also use the phase.

#### 2.1.1. Centroids (MSC) and Dynamic Range per Band (DRB)

Centroids provide cues about the acoustic frequency that represents the central energy or the energy center at each modulation band. To obtain MSC, MS modulus is reduced to an absolute number of modulation bands usually ranging from 4 to 26, each containing information about the modulation energy in that band along the acoustic frequency axis. Once the reduced MS is computed, centroids are calculated following the expression

(1) $$\begin{array}{c} \text{MSC}\left(\;f_{m}\;\right)=\frac{\sum_{a}f_{a}\cdot \left|E\left(f_{a},f_{m}\right)\right|}{f_{pitch}\cdot \sum_{a}\left|E\left(f_{a},f_{m}\right)\right|}, \end{array}$$

where *f*_*a*_ and *f*_*m*_ represent the central frequency of the acoustic and modulation bands, respectively, and *f*_pitch_ is the pitch frequency.

**Figure 2:**
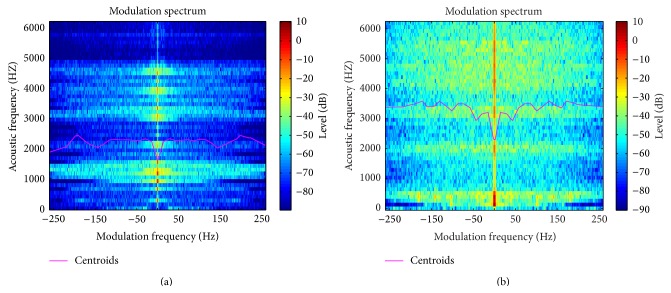
MS centroids of a normal voice (a) and a pathological voice (b) of a patient with gastric reflux, keratosis, and laryngocele.

As a matter of example, Figure 2 depicts a representation of MSC extracted from a MS.

Once MS is reduced to a small number of modulation bands, the dynamic range is calculated for every band (DRB) as the difference between the highest and the lowest levels in the band. These parameters provide information about the flatness of the MS depending on the modulation frequency.

#### 2.1.2. Low Modulation Ratio (LMR)

**Figure 3:**
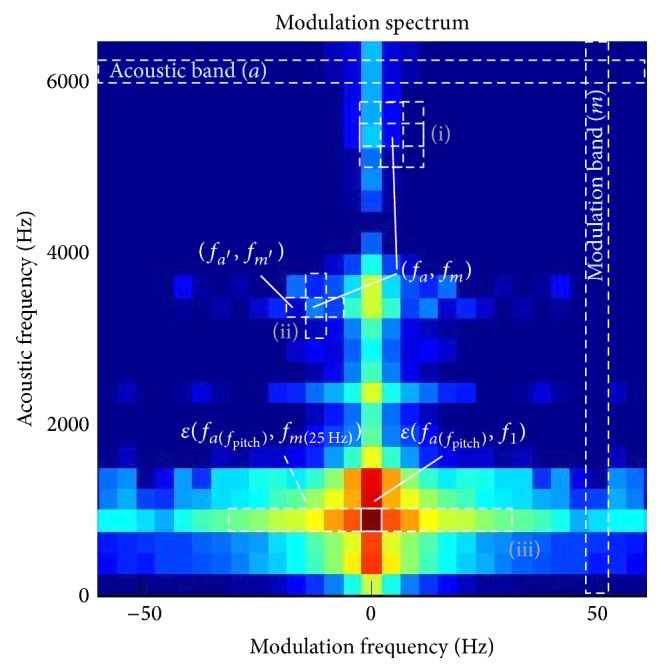
Points of the MS matrix used to obtain MSH (i), MSW (ii), and LMR (iii).

LMR, expressed in dB, is the ratio between energy in the first modulation band *ɛ*(*f*_*a*(*f*_pitch_)_, *f*_1_) at acoustic frequency *f*_pitch_ and the global energy in all modulation bands covering at least from 0 to 25 Hz at acoustic frequency *f*_pitch_, *ɛ*(*f*_*a*(*f*_pitch_)_, *f*_*m*(25 Hz)_). Its calculation is carried out according to the following expressions. These bands are represented in Figure 3:

(2) $$\begin{array}{c} \text{LMR}=10\cdot \log\left(\;\frac{ɛ\left(f_{a\left(f_{pitch}\right)},f_{1}\right)}{ɛ\left(f_{a\left(f_{pitch}\right)},f_{m\left(25\;Hz\right)}\right)}\;\right) \end{array}$$

being

(3) $$\begin{array}{c} ɛ\left(\;f_{a},f_{k}\;\right)=\overset{k}{\underset{m=1}{\sum }}{\left|\;E\left(\;f_{a},f_{m}\;\right)\;\right|}^{2}, \end{array}$$

where *a*(*f*_pitch_) is the index of the acoustic band including pitch frequency and *m*(25 Hz), the index of the modulation band including 25 Hz.

The 0–25 Hz band has been selected to represent all possible cases of tremor and low frequency modulations around pitch frequency [52, 53].

#### 2.1.3. Contrast and Homogeneity

Representing MS (modulus or phase) as two-dimensional images let observe that pathological voices usually have more complex distributions. Images related to normal voices are frequently more homogeneous and present less contrast, as can be seen in Figure 1. Accordingly, Homogeneity and Contrast are used as two MS features since they provide information about the existence of voice perturbations.

Homogeneity is computed using the Bhanu method described by the following expression, as stated in [54]:

(4) $$\begin{array}{c} \text{MSH}=\underset{a}{\sum }\underset{m}{\sum }{\left[\;E\left(f_{a},f_{m}\right)-{\bar{E\left(f_{a},f_{m}\right)}}_{3\times 3}\;\right]}^{2}, \end{array}$$

with MSH being the MS Homogeneity value; *E*(*f*_*a*_, *f*_*m*_) the modulation spectra computation (modulus or phase) at point (*f*_*a*_, *f*_*m*_); and ${\bar{E(f_{a},f_{m})}}_{3\times 3}$ the average value in a 3 × 3 window centered at the same point.

Contrast is computed using a variation of the Weber-Fechner contrast relationship method described by the following expression as stated in [54]:

(5) $$\begin{array}{c} \text{MSW}\left(\;f_{a},f_{m}\;\right)=\underset{a^{\prime }}{\sum }\underset{m^{\prime }}{\sum }C_{f_{a},f_{m}}, \end{array}$$

where

(6) $$\begin{array}{c} C_{f_{a},f_{m}}=\frac{\left|E\left(f_{a},f_{m}\right)-E\left(f_{a^{\prime }},f_{m^{\prime }}\right)\right|}{\left|E\left(f_{a},f_{m}\right)+E\left(f_{a^{\prime }},f_{m^{\prime }}\right)\right|} \end{array}$$

representing (*f*_*a*′_, *f*_*m*′_) the vertical and horizontal adjacent points to (*f*_*a*_, *f*_*m*_). The global MSW is considered the sum of all points in MSW(*f*_*m*_, *f*_*a*_) divided by the total number of points to normalize.

The MS used to calculate MSH and MSW at each point of the matrix is represented in Figure 3.

#### 2.1.4. Dispersion Parameters

As MS differs from normal to pathological voices, changes in the histograms of MS modulus reflect the effects of a dysfunction in a patient's voice. A short view to the MS permits to observe that voices with high *G* and *R* traits usually have a larger number of points with levels above the average value of |*E*(*f*_*m*_, *f*_*a*_)|. The level of these points can be interpreted as the dispersion of the energy present in the central modulation band (0 Hz) towards side bands respecting the case of a normal voice.

**Figure 4:**
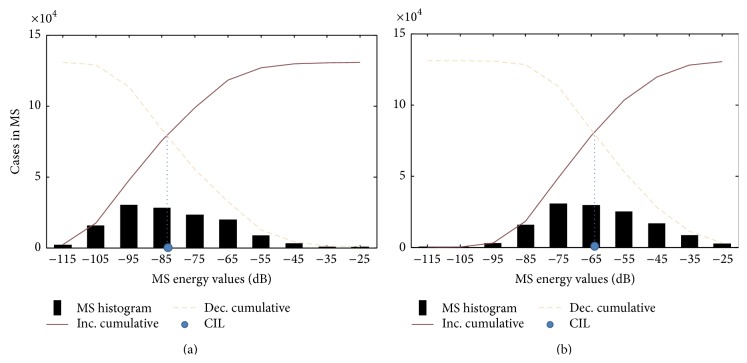
CIL calculation in a normal voice (top) and a pathological voice (bottom) diagnosed of bilateral laryngeal tuberculosis.

With this in mind, three Morphological Parameters are proposed to measure such dispersion effect:* Cumulative Intersection Level (CIL)*,* Normalized Number of Points above Linear Average (PALA)*, and* Ratio of Points above Linear Average (RALA)*. CIL is the intersection between the histogram increasing and decreasing cumulative curves. Histogram is processed from MS modulus in logarithmic units (dB). As shown in Figure 4, CIL tends to be higher in pathological than in healthy voices. In that case, the difference is 19 dB. On the other hand, PALA is the number of points in MS modulus which are above average (linear units) divided by the total number of points of MS. RALA is quite similar to PALA but in this case it represents the ratio of points in MS modulus which are over the average and the number of points which are above this average instead of the total number of points in *E*(*f*_*a*_, *f*_*m*_). Calculation of PALA and RALA is detailed in the following expressions:

(7) PALANANT,RALA=NANB

being

(8) NA∑fa∑fmγfa,fm,NB=∑fa∑fm1−γfa,fm,γfa,fm=1Efa,fm≥E¯0Efa,fm<E¯,

where $\bar{\left|E\right|}$ is the MS modulus average, NA the number of points above $\bar{\left|E\right|}$, NB the number of points below $\bar{\left|E\right|}$, and NT the total number of points in *E*(*f*_*a*_, *f*_*m*_).

**Figure 5:**
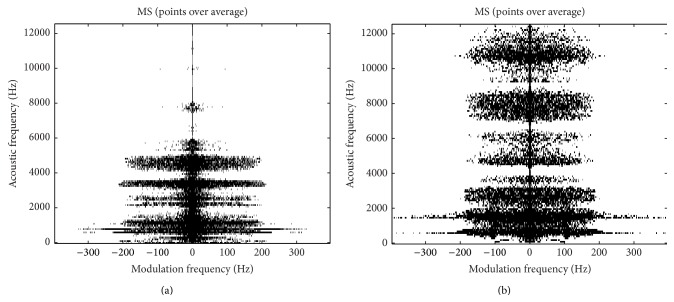
Points above (black) and below (white) modulus average in MS for a normal voice (a) PALA = 0.11, RALA = 0.12, and a pathological voice due to bilateral laryngeal tuberculosis (b) PALA = 0.21, RALA = 0.27.

In the cases in which the number of points above linear average increases, the difference between PALA and RALA increases too as the denominator in PALA stays constant and the denominator in RALA decreases.  Figure 5 represents these points in a healthy and a pathological voice. It is noticeable that, as expected, the MS of dysphonic voices presents more points above the modulus average.

## 3. Experimental Setup

### 3.1. Database

The Kay Elemetrics Voice Disorders Database recorded by the Massachusetts Eye and Ear Infirmary Voice Laboratory (MEEI) was used for this study [55] due to its commercial availability. The database contains recordings of the phonation of the sustained vowel /*ah*  :  / (53 normal, 657 pathological) and utterances corresponding to continuous speech during the reading of the “Rainbow passage” (53 normal, 661 pathological). The sample frequency of the recordings is 25 kHz with a bit depth of 16 bits. From the original amount of speakers recorded in the database, a first corpus of 224 speakers was selected according to the criteria found in [56] being named henceforward as the* original subset*. The utterances corresponding to the sustained vowel and the continuous speech recordings were used to rate *G* and *R* for each patient according to the GRBAS scale. The degree of these traits has been estimated three times by two speech therapists. One of them evaluated the whole database once, and the second one performed the assessment twice in two different sessions. Regarding this study, only the sustained vowels are considered. With the aim of obtaining more consistent labels, two reduced subsets of 87 and 85 audio files for *G* and *R*, respectively, were considered. Those files are chosen from the initial corpus of 224 recordings on the basis of selecting only those whose labeling was in a total agreement for the three assessments making up the *G* and *R agreement subsets*. This reduction was performed to avoid modeling inter/intraraters variability inherent to the process of assigning perceptual labels to each speaker. In any case, all tests were performed for the three subsets to provide evidences about such reduction. Some statistics of database are shown in Table 1.

With the aim of sharing relevant information and to promote a more reliable comparison of techniques and results, the names of the recordings extracted from MEEI corpus that were used for this study along with their respective *G* and *R* levels are included in Appendix, Table 6.

### 3.2. Methodology

One of the purposes of this work is to test a new source of parameters to characterize voice perturbations by replicating clinician's *G* and *R* perceptual evaluations. So as to quantify the contribution of this new approach, a baseline parameterization has been established to compare with the novel one. Consequently, all tests are performed using the parameters of the baseline system (MFCCs) and the MS* Morphological Parameters*. A large number of tests were accomplished to find the best setting, modifying the number of centroids or the frame duration among other degrees of freedom.

**Figure 6:**
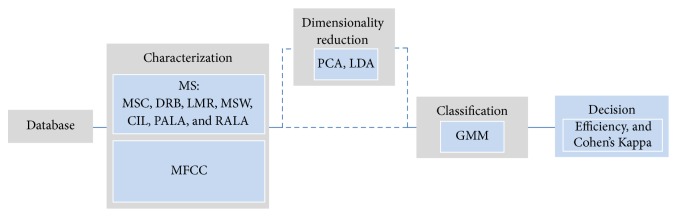
Outline of the automatic detector presented in the paper.

The methodology employed in this paper is shown in Figure 6, while each one of its stages is explained next. Basically, it is the classical supervised learning arrangement, which can be addressed using either classification or regression techniques. For the sake of simplicity and to concentrate on the novel parameterization approach, a simple Gaussian Mixture Model (GMM) classification back-end was employed to recognize the presence of the perturbations in the voice signal which presumably would produce high levels of *G* and *R* during perceptual analysis.

#### 3.2.1. Characterization

Two parameterization approaches are considered in this study: MFCCs and MS Morphological Parameters. The MFCCs are the ground of the baseline system and were used for comparison due to their wide use in speech technology applications.

The MFCCs are calculated following a method based on the human auditory perception system. The mapping between the real frequency scale (Hz) and the perceived frequency scale (mels) is approximately linear below 1 kHz and logarithmic for higher frequencies. Such mapping converts real into perceived frequency. In this work MFCCs are estimated using a nonparametric FFT-based approach. Coefficients are obtained by calculating the Discrete Cosine Transform (DCT) over the logarithm of the energy in several frequency bands. The bandwidth of the critical band varies according to the perceived frequency. Each band in the frequency domain is bandwidth dependant of the filter central frequency. The higher the frequency is, the wider the bandwidth is. To obtain these parameters, a typical setup of 30 triangular filters and cepstral mean subtraction was used. Their computation is carried out over speech segments framed and windowed using Hamming windows overlapped 50%. Duration of frames oscillates from 20 to 100 ms in 20 ms steps. For the sake of comparison the number of MFCCs ranges from 10 to 22 coefficients. 0'th order cepstral coefficient is removed.

Regarding the MS Morphological Parameters, each signal is also framed and windowed using Hamming windows overlapped 50%. The window lengths are varied in the range of 20–200 ms in 20 ms steps. The feature vector extracted from MS is composed of the following: MSC, DRB, LMR, MSW, MSH, CIL, PALA, and RALA. The number of bands to obtain centroids and dynamic range features is varied in the range of [6,22] with a step size of 2. Considering that MSW and MSH provide two features each (one for modulus and other for phase), the feature vector corresponding to each frame ranges from 20 to 44 values before using data reduction techniques. Both, coherent and noncoherent modulation (Hilbert envelope) were used for testing separately. Acoustic frequency span [0–12.5 kHz] is divided into 128 bands and maximum modulation frequency varied from 70 to 500 Hz to allow different configurations during tests.

In addition, first derivative (Δ) and second derivative (ΔΔ), representing the speed and acceleration in the changes of every characteristic, are added to the features in order to include interframe attributes [46]. The calculation of Δ and ΔΔ was carried out employing finite impulse response filters using a length of 9 samples to calculate Δ and 3 in the case of ΔΔ.

All these features are used to feed a subsequent classification phase in two different ways depending on the test: some experiments are accomplished using features as they are obtained, and others use a reduced version to relieve* the curse of dimensionality* effect.

In the dimensionality reduction stage, PCA [57] and LDA [58] techniques are used varying the dimension of the feature vectors used for classification. In the case of LDA, all feature vectors are reduced to a 3-dimensional space. Concerning PCA, reduction ranges from 80 to 95%. With respect to these techniques, only the training data set is used to obtain the models which are employed to reshape all the data: training and test data sets. This process is repeated for every iteration of the GMM training-test process carried out for validation. The dimensionality reduction is applied for both MS Morphological Parameters and MFCCs features with and without derivatives separately.

#### 3.2.2. Validation

Following the characterization, a* Leave-One-Out (LOO)* cross-validation scheme [33] was used for evaluating the results. On this scheme one file is considered for testing and the remaining files of the database are used as training data, generating what is called a* fold*. As a result, there are as many* folds* as number of files, and each of them will provide a classification accuracy. The global result for a certain parameterization experiment is the average of the results in all folds. In spite of having a higher computational cost, this cross-validation technique has been selected instead of other less computationally costly such as *k*-folds [59] due to its suitability in view of the reduced number of recordings contained in the* agreement subsets*.

#### 3.2.3. Classification

The features extracted during the parameterization stage are used to feed the classifier, which is based on the* Gaussian Mixture Model* (GMM) paradigm. Having a data vector **x** of dimension *d* resulting from the parameterization stage, a GMM is a model of the probability density function defined as a finite mixture of *g* multivariate Gaussian components of the form:

(9) $$\begin{array}{c} p\left(\;x\;|\;\Theta_{i}\;\right)=\overset{g}{\underset{r=1}{\sum }}\lambda_{r}N\left(\;x;\mu_{r},\Sigma_{r}\;\right), \end{array}$$

where ***λ***_*r*_ are scalar mixture weights, *𝒩*(·) are Gaussian density functions with mean ***μ***_*r*_ of dimension *d* and covariances Σ_*r*_ of dimension *d* × *d*, and Θ_*i*_ = {***λ***_*r*_, ***μ***_*r*_, Σ_*r*_}|_*r*=1_^*g*^ comprises the abovementioned set of parameters that defines the class to be modeled. Thus, for each class Θ_*i*_ to be modeled (i.e., values of the *G* and *R* perceptual levels: 0, 1, 2, or 3), a GMM is trained. Θ_*i*_ is estimated using the* expectation-maximization* algorithm (EM) [60]. The final decision about the class that a vector belongs to is taken establishing for each pair of classes *i*, *j* a threshold Γ over the likelihood ratio (LR), that in the logarithmic domain is given by

(10) $$\begin{array}{c} \text{LR}=\log\left(\;p\left(\;x\;|\;\Theta_{i}\;\right)\;\right)-\log\left(\;p\left(\;x\;|\;\Theta_{j}\;\right)\;\right). \end{array}$$

The threshold Γ is fixed at the Equal Error Rate (ERR) point.

In this stage, the number of Gaussian components of the GMM was varied from 4 to 48. The assessment of the classifier was performed by means of efficiency and Cohen's Kappa Index (*κ*) [61]. This last indicator provides information about the agreement between the results of the classifier and the clinician's perceptual labeling.

## 4. Results

The best results obtained for each type of test can be observed in Table 2, which disposes the outcomes taking into account the type of characterization, dimensionality reduction, and database subset used. All tests were performed using the aforementioned sets of the database with and without PCA and LDA techniques.  Table 3 shows the outcomes adding first and second derivative to the original parameterizations before dimensionality reduction. All results are expressed in terms of efficiency and Cohen's Kappa Index. For the sake of simplicity, only results obtained with the third labeling of the* original subset* are shown, corresponding to columns *G*3 and *R*3 in Appendix, Table 6.

Concerning *G* trait, absolute best results (81.6%) are obtained in the* agreement database*, using MS + Δ in 140 ms frames, 22 centroids, Hilbert envelope, 240 Hz as max. modulation frequency, dimensionality reduction through PCA (93% reduction), and 4 GMM. Respecting MFCC, best results are obtained using MFCCs + Δ + ΔΔ, 22 coefficients, PCA, 20 ms frames, and 8 GMM.

Relating to *R*, as expected, absolute best results (84.7%) are also obtained in the agreement database using MS + Δ + ΔΔ calculated in 100 ms frames, 14 centroids, Hilbert envelope, 240 Hz as max. modulation frequency, dimensionality reduction through LDA, and 16 GMM. Respecting MFCC, best results are obtained using MFCCs + Δ + ΔΔ, 22 coefficients, PCA, 20 ms frames, and 48 GMM.

Table 4 shows confusion matrices for MFCC and MS Morphological Parameters as the sum of the confusion matrices obtained at each of the test folds. They are calculated using the mentioned configurations that leaded to the best results.

## 5. Conclusion and Discussions

This study presents a new set of parameters based on MS being developed to characterize perturbations of the human voice. The performance of these parameters has been tested with an automatic system that emulates a perceptual assessment according to the *G* and *R* features of the GRBAS scale. The proposed automatic system follows a classical supervised learning setup, based on GMM. The outcomes have been compared to those obtained with a baseline setup using the classic MFCCs as input features. Dimensionality reduction methods as LDA and PCA have been applied to mitigate* the curse of dimensionality* effects induced by the size of the corpus used to train and validate the system. Best results are obtained with the proposed MS parameters, providing 81.6% and 84.7% of efficiency and 0.73 and 0.76 Cohen's Kappa Index for *G* and *R*, respectively, in the* agreement subset*. Having in mind Altman interpretation of Cohen's index [62], shown in Table 5, the agreement can be considered “good”, almost “excellent.” Likewise, most errors raised by the system correspond with adjacent classes, as it can be deduced from the confusion matrices represented in Table 4. It is noticeable that in many cases the second class (level 1 in traits *G* and *R*) is not detected properly and the main reason may be the lack of subjects of class 2 (level 1 in *G* and *R*) in the used corpus. The fact that GMM classifiers were trained with a poor quantity of class 2 frames with respect to the other classes explains the higher percentage of errors obtained for this class. In order to solve this problem in future works it might be necessary to use classification techniques for imbalanced data [63]. Another possible reason for the mismatching of intermediate classes (*G* and *R* equal to 1 or 2) is that these are the less reliable levels in GRBAS perceptual assessment as it was described by de Bodt et al. [5].

In reference to the outcomes obtained with features without dimensionality reduction, results are better for the* agreement subsets* using MS Morphological Parameters. Moreover, when applying LDA to the MS feature space, an absolute improvement of a 9% is obtained for *R* in comparison to MFCCs, leading to the best absolute outcome obtained and denoting that the MS Morphological Parameters are in some sense linearly separable. As a starting point, most of the* agreement subset* tests were performed with what we have called* the original subset* (224 files) using the three available label groups separately: one of them generated by one of the speech therapists and the other two created by the other specialist in two different sessions. In these cases, in spite of having a higher number of files and a more class-balanced database, results barely exceed 60% of efficiency. This demonstrates that the consistency of the database labeling (i.e., removing the noise introduced during the labeling process due to intra- and interrater's variability) is crucial to obtain more accurate results. An interesting conclusion is that further studies should utilize only consistent labels obtained in agreement with several therapists and in different evaluation sessions.

In order to search for some evidences proving that the selected cross-validation technique is not influencing the results by producing corpus-adjusted trained models, most of the tests are launched again using a 6-fold cross-validation technique as a prospecting experiment. Almost the same maximum efficiencies were obtained in all cases with a difference of around ±1%, suggesting that the selected cross-validation technique is not producing corpus-adjusted trained models.

Regarding the use of derivatives Δ and ΔΔ, they improve performance mainly when using MFCCs in 20 ms frames for *G* trait. This suggests that derivatives provide relevant extra information related to the short term variations occurred in pathological voices [64]. In the rest of the cases the improvements are limited; therefore, the influence of derivatives in *G* and *R* detection systems should be studied in detail in the future work.

Comparing this work with other studies mentioned in Section 1, results with MFCCs are coherent with these obtained in [17, 21, 36], although methodologies followed in them are different to the one proposed in this study. As it is stated in Section 1, previous studies seldom exceed 75% efficiency. Taking into account *G* and *R* traits, only [19, 20] surpass that value achieving 80% for *G* trait.

Despite the promising results, an accurate comparison with the studies found in the state of the art is difficult since, as stated in [65], different works tend to use different types of corpus and methodologies, and results are unfortunately dependant of the corpus used for training and validation. Furthermore, those cases on which different studies utilize the same database, labeling is usually different. In this sense, the definition of a standardized database with a consistent and known labeling would lead to comparable results. For this reason, with the aim of providing the scientific community with a benchmark labeling and to promote a more solid comparative estimate of future techniques and studies, the labeling of the *G* and *R* features used on this work has been included in Table 6. Despite its known limitations [65], the fact that MEEI database is commercially available for researchers is also an advantage in this sense.

On the other hand, other approaches such as [11, 23] have already demonstrated that MS is a good source of information to detect pathological voices or to perform an automatic pathology classification. The main difference with respect to Markaki's approach is that in this study MS is used to evaluate the speech according to a 4-level scale in two different features of the speech: Grade and Roughness. On the other hand, the parameters used in the present study are less abstract and have an easier physical interpretation, opening the possibility of using them in a clinical setting.

In spite of the good figures, MS has a weakness which could make it a nonviable parameterization in some applications: computational cost. Depending on the configuration and frequency margins, to calculate a MS matrix can take around 400 times more than to calculate MFCCs on the same signal frame.

Regarding the future work, all MS parameters must be studied and adjusted separately to find the adequate frequency margins of operation to optimize results. In addition, the use of the proposed MS Morphological Parameters in combination with some other features such as complexity and noise measurements or cepstral-based coefficients to characterize GRBAS traits would be advisable. Moreover, the study of regression methods like Support Vector Regression [66] and other feature selection techniques such as Least Absolute Shrinkage and Selection Operator (LASSO) [67] is of interest. In respect of the classification stage, the stratification of the speakers according to her/his sex, age, or emotional state could increase performance as suggested in [68]. For this purpose, a priori categorization of speaker's characteristics using hierarchical methods might be used to simplify the statistical models behind to automatically assess the quality of speech.

Summarizing, results suggest that the proposed MS Morphological Parameters are an objective basis to help clinicians to assess Grade and Roughness according the GRBAS scale, reducing uncertainty and making the assessment easier to replicate. It would be advisable to study the synthesis of a new parameter combining the proposed MS Morphological Parameters, being suitable for therapists and physicians. In view of the experiments carried out in this work, there are evidences that suggest that the use of these parameters provides better results than the classic MFCCs, traditionally used to characterize voice signals. On the other hand, its main drawback is the initial difficulty of applying the proposed MS-based parameters to the study of running speech.

## Figures and Tables

**Table 1:** Subsets statistics.

Subset name	Number of subjects		Age range		Average age	
	Female	Male	Female	Male	Female	Male
Original (226 files)	90	134	21–52	26–59	35.8 ± 8.2	39.9 ± 9.1
Agreement-*G* (87 files)	52	35	24–52	26–58	36.6 ± 7.6	39.5 ± 9.7
Agreement-*R* (85 Files)	51	34	22–52	26–58	35.4 ± 7.6	37.9 ± 9.2

**Table 2:** Results expressed as efficiency ± confidence interval and Cohen's Kappa Index using MFCCs and MS features.

Features	Original subset				Agreement subset			
	*G*		*R*		*G*		*R*	
	Efficiency (%)	*κ*	Efficiency (%)	*κ*	Efficiency (%)	*κ*	Efficiency (%)	*κ*
MFCC	54.5 ± 6.5	0.37	53.1 ± 6.5	0.51	75.9 ± 9.0	0.64	76.5 ± 9.0	0.64
MFCC + PCA	56.3 ± 6.5	0.39	52.2 ± 6.6	0.31	78.2 ± 8.7	0.67	74.1 ± 9.3	0.60
MFCC + LDA	45.5 ± 6.5	0.27	48.2 ± 6.6	0.29	65.5 ± 10.0	0.48	68.3 ± 9.9	0.50

MS	60.3 ± 6.4	0.45	54.9 ± 6.5	0.36	81.6 ± 8.1	0.72	76.5 ± 9.0	0.63
MS + PCA	58.5 ± 6.5	0.43	58.0 ± 6.5	0.41	79.3 ± 8.5	0.69	78.8 ± 8.7	0.68
MS + LDA	58.9 ± 6.4	0.44	59.8 ± 6.4	0.43	81.6 ± 8.1	0.72	83.5 ± 7.9	0.74

**Table 3:** Results expressed as efficiency ± confidence interval and Cohen's Kappa Index for MFCCs and MS features including Δ and ΔΔ.

Features	Dimensionality reduction	Agreement subset			
		*G*		*R*	
		Efficiency (%)	*κ*	Efficiency (%)	*κ*
MFCC + Δ	PCA	78.2 ± 8.7	0.67	74.1 ± 9.3	0.60
	LDA	72.4 ± 9.4	0.58	58.8 ± 10.5	0.35
MFCC + Δ + ΔΔ	PCA	**80.5** ± **8.3**	**0.71**	**77.7** ± **8.8**	**0.66**
	LDA	72.4 ± 9.4	0.58	62.4 ± 10.3	0.41

MS + Δ	PCA	**81.6** ± **8.1**	**0.73**	80.0 ± 8.5	0.69
	LDA	80.5 ± 8.3	0.72	81.2 ± 8.3	0.71
MS + Δ + ΔΔ	PCA	79.3 ± 8.5	0.63	80.0 ± 8.5	0.70
	LDA	80.5 ± 8.3	0.71	**84.7** ± **7.7**	**0.76**

**Table 4:** Confusion matrices related to absolute best results in MS parameters and MFCCs. *G*_*T*_ and *R*_*T*_ are target labels while *G*_*P*_ and *R*_*P*_ are predicted labels.

Grade					Roughness				
	*G*_*P*_0	*G*_*P*_1	*G*_*P*_2	*G*_*P*_3		*R*_*P*_0	*R*_*P*_1	*R*_*P*_2	*R*_*P*_3
MS Morphological Parameters									
*G*_*T*_0	** 28**	1	0	1	*R*_*T*_0	** 38**	1	0	0
*G*_*T*_1	3	** 3**	1	0	*R*_*T*_1	3	** 1**	2	0
*G*_*T*_2	1	1	** 11**	1	*R*_*T*_2	1	0	** 13**	1
*G*_*T*_3	3	0	4	** 29**	*R*_*T*_3	3	1	1	** 20**

MFCCs									
*G*_*T*_0	** 27**	0	2	1	*R*_*T*_0	** 35**	0	1	3
*G*_*T*_1	2	** 0**	5	0	*R*_*T*_1	3	** 0**	3	0
*G*_*T*_2	0	0	** 13**	1	*R*_*T*_2	1	0	** 9**	5
*G*_*T*_3	0	0	6	** 30**	*R*_*T*_3	1	0	2	** 22**

**Table 5:** Altman interpretation of Cohen's index.

κ	Agreement
≤0.20	Poor
0.21–0.40	Fair
0.41–0.60	Medium
0.61–0.80	Good
0.81–1.00	Excellent

**Table 6:** Subsets labeling.

File	*G*1	*G*2	*G*3	*R*1	*R*2	*R*3	File	*G*1	*G*2	*G*3	*R*1	*R*2	*R*3	File	*G*1	*G*2	*G*3	*R*1	*R*2	*R*3	File	*G*1	*G*2	*G*3	*R*1	*R*2	*R*3
alb18an	2	3	3	2	3	3	gpc1nal	** 0**	** 0**	** 0**	** 0**	** 0**	** 0**	lba15an	2	0	0	1	0	0	pmc26an	2	3	3	** 2**	** 2**	** 2**
amc14an	1	3	3	1	3	3	gsb11an	** 3**	** 3**	** 3**	** 3**	** 3**	** 3**	lba24an	2	0	0	1	0	0	pmd25an	2	3	2	2	3	2
aos21an	** 3**	** 3**	** 3**	** 3**	** 3**	** 3**	gxl21an	1	0	0	1	0	0	ldp1nal	1	0	0	** 0**	** 0**	** 0**	pmf03an	2	1	1	2	1	1
axd19an	** 0**	** 0**	** 0**	** 0**	** 0**	** 0**	gxt10an	** 3**	** 3**	** 3**	** 3**	** 3**	** 3**	les15an	** 3**	** 3**	** 3**	3	0	0	rcc11an	2	3	3	** 2**	** 2**	** 2**
axh1nal	** 0**	** 0**	** 0**	** 0**	** 0**	** 0**	gzz1nal	1	0	0	1	0	0	lgm01an	1	0	0	1	0	0	rhg1nal	0	1	0	0	1	0
axt13an	** 1**	** 1**	** 1**	1	0	1	hbl1nal	1	0	0	1	0	0	ljh06an	** 2**	** 2**	** 2**	** 2**	** 2**	** 2**	rhm1nal	** 0**	** 0**	** 0**	** 0**	** 0**	** 0**
bah13an	1	3	2	1	3	2	hjh07an	2	3	3	2	3	3	ljs31an	2	1	1	1	1	0	rhp12an	2	3	2	2	3	2
bef05an	2	3	3	2	0	0	hlm24an	1	2	2	1	2	2	lla1nal	1	0	0	** 0**	** 0**	** 0**	rjf22an	2	3	3	2	3	3
bjb1nal	** 0**	** 0**	** 0**	** 0**	** 0**	** 0**	hxi29an	1	3	2	1	3	2	llm22an	3	3	2	3	3	2	rjl28an	3	3	2	2	3	2
bjv1nal	** 0**	** 0**	** 0**	** 0**	** 0**	** 0**	hxl58an	1	0	0	1	0	0	lmv1nal	1	0	0	1	0	0	rjr15an	** 1**	** 1**	** 1**	** 1**	** 1**	** 1**
bkb13an	1	0	1	2	0	1	jaf1nal	** 0**	** 0**	** 0**	** 0**	** 0**	** 0**	lmw1nal	1	0	0	** 0**	** 0**	** 0**	rjs1nal	** 0**	** 0**	** 0**	** 0**	** 0**	** 0**
blb03an	2	3	2	2	3	2	jan1nal	** 1**	** 1**	** 1**	** 1**	** 1**	** 1**	lnc11an	1	0	0	** 0**	** 0**	** 0**	rjz16an	1	0	0	1	0	0
bpf03an	1	2	1	1	2	1	jap02an	2	1	1	2	0	1	lrd21an	1	0	0	** 0**	** 0**	** 0**	rmb07an	2	3	3	2	3	3
bsg13an	** 1**	** 1**	** 1**	** 1**	** 1**	** 1**	jap1nal	** 0**	** 0**	** 0**	** 0**	** 0**	** 0**	lvd28an	2	3	2	** 2**	** 2**	** 2**	rpj15an	** 3**	** 3**	** 3**	** 3**	** 3**	** 3**
cac10an	2	3	3	2	0	0	jcc10an	** 2**	** 2**	** 2**	** 2**	** 2**	** 2**	lwr18an	1	3	3	1	0	0	rpq20an	2	3	3	2	3	3
cad1nal	** 0**	** 0**	** 0**	** 0**	** 0**	** 0**	jcr01an	** 3**	** 3**	** 3**	** 3**	** 3**	** 3**	lxc01an	** 2**	** 2**	** 2**	2	0	0	rtl17an	1	0	1	1	0	1
cak25an	2	1	1	2	1	1	jeg1nal	** 0**	** 0**	** 0**	** 0**	** 0**	** 0**	lxc06an	** 3**	** 3**	** 3**	** 3**	** 3**	** 3**	rwc23an	2	3	3	2	3	3
ceb1nal	** 0**	** 0**	** 0**	** 0**	** 0**	** 0**	jeg29an	2	3	2	2	0	0	lxr15an	3	3	2	3	3	2	rxm15an	1	0	0	1	0	0
cls31an	2	1	1	2	1	1	jfg08an	** 3**	** 3**	** 3**	** 3**	** 3**	** 3**	mab06an	2	1	1	2	1	1	rxp02an	1	3	3	1	3	3
cma06an	1	2	1	1	2	1	jfn21an	3	2	2	3	2	2	mam08an	2	3	3	2	3	3	sac10an	2	3	3	2	3	3
cmr06an	** 0**	** 0**	** 0**	** 0**	** 0**	** 0**	jhw29an	2	1	1	2	1	1	mam1nal	** 0**	** 0**	** 0**	** 0**	** 0**	** 0**	sae01an	1	0	0	1	0	0
crm12an	** 3**	** 3**	** 3**	3	2	2	jkr1nal	1	0	0	1	0	0	mas1nal	** 0**	** 0**	** 0**	** 0**	** 0**	** 0**	sav18an	** 3**	** 3**	** 3**	** 3**	** 3**	** 3**
ctb30an	2	2	1	2	1	1	jld24an	2	3	2	2	3	2	mcb1nal	1	0	0	1	0	0	sbf11an	** 0**	** 0**	** 0**	** 0**	** 0**	** 0**
daj1nal	** 0**	** 0**	** 0**	** 0**	** 0**	** 0**	jls11an	2	1	1	2	1	1	mcw21an	2	1	1	2	1	1	scc15an	** 3**	** 3**	** 3**	3	0	1
dap17an	2	2	1	2	2	1	jmc18an	1	0	0	1	0	0	mec06an	2	0	0	2	0	0	sck1nal	1	0	0	1	0	0
das30an	1	—	1	1	—	1	jmc1nal	1	0	0	1	0	0	mec28an	2	2	1	1	2	1	sct1nal	1	0	0	1	0	0
dbf18an	2	2	1	2	2	1	jpp27an	** 3**	** 3**	** 3**	3	0	0	mfc20an	** 3**	** 3**	** 3**	2	3	3	seb1nal	1	0	0	1	0	0
dfp1nal	** 0**	** 0**	** 0**	** 0**	** 0**	** 0**	jrf30an	1	0	0	1	0	0	mfm1nal	1	0	0	1	0	0	sec02an	2	0	1	2	0	1
djg1nal	** 0**	** 0**	** 0**	** 0**	** 0**	** 0**	jth1nal	1	0	0	** 0**	** 0**	** 0**	mju1nal	** 0**	** 0**	** 0**	** 0**	** 0**	** 0**	sef10an	1	0	0	1	0	0
djp04an	** 2**	** 2**	** 2**	** 2**	** 2**	** 2**	jtm05an	1	2	1	1	2	1	mpb23an	** 3**	** 3**	** 3**	** 3**	** 3**	** 3**	seg18an	2	1	1	2	1	1
dma1nal	** 0**	** 0**	** 0**	** 0**	** 0**	** 0**	jxc1nal	1	0	0	** 0**	** 0**	** 0**	mpf25an	1	2	1	1	2	1	sek06an	2	2	1	2	2	1
dmc03an	2	3	2	2	0	0	jxc21an	2	0	0	2	0	0	mps09an	2	0	1	2	0	1	shd04an	** 3**	** 3**	** 3**	** 3**	** 3**	** 3**
dmp04an	** 2**	** 2**	** 2**	** 2**	** 2**	** 2**	jxd30an	** 1**	** 1**	** 1**	0	1	1	mrb11an	1	3	2	1	3	2	sis1nal	** 0**	** 0**	** 0**	** 0**	** 0**	** 0**
drc15an	** 2**	** 2**	** 2**	2	0	0	jxf11an	** 3**	** 3**	** 3**	** 3**	** 3**	** 3**	mrc20an	2	3	2	2	3	2	sjd28an	** 1**	** 1**	** 1**	** 1**	** 1**	** 1**
dsc25an	** 3**	** 3**	** 3**	3	0	0	kab03an	1	0	0	1	0	0	mwd28an	2	3	2	2	3	2	slc1nal	2	0	0	1	0	0
dsw14an	2	—	1	2	—	1	kac07an	1	0	0	1	0	0	mxb1nal	** 0**	** 0**	** 0**	** 0**	** 0**	** 0**	slc23an	2	0	1	1	0	1
dvd19an	** 3**	** 3**	** 3**	** 3**	** 3**	** 3**	kan1nal	** 0**	** 0**	** 0**	** 0**	** 0**	** 0**	mxc10an	** 2**	** 2**	** 2**	** 2**	** 2**	** 2**	slg05an	1	0	0	1	0	0
dwk04an	** 2**	** 2**	** 2**	** 2**	** 2**	** 2**	kcg23an	2	2	1	2	2	1	mxn24an	1	0	0	1	0	0	sma08an	** 3**	** 3**	** 3**	** 3**	** 3**	** 3**
dws1nal	** 0**	** 0**	** 0**	** 0**	** 0**	** 0**	kcg25an	1	0	0	1	0	0	mxz1nal	1	0	0	** 0**	** 0**	** 0**	sws04an	3	2	2	3	2	2
eab27an	** 3**	** 3**	** 3**	** 3**	** 3**	** 3**	kdb23an	** 2**	** 2**	** 2**	** 2**	** 2**	** 2**	nfg08an	2	2	1	1	2	1	sxv1nal	1	0	0	1	0	0
eas11an	1	2	1	1	2	1	kjb19an	2	3	3	2	3	3	njs06an	2	2	1	2	2	1	tab21an	** 3**	** 3**	** 3**	** 3**	** 3**	** 3**
eas15an	** 3**	** 3**	** 3**	** 3**	** 3**	** 3**	klc06an	2	3	3	2	0	0	njs1nal	1	0	0	1	0	0	tdh12an	2	2	1	2	2	1
edc1nal	1	0	0	1	0	0	klc09an	2	3	2	2	3	2	nkr03an	1	0	0	1	0	0	tlp13an	** 2**	** 2**	** 2**	** 2**	** 2**	** 2**
eec04an	** 3**	** 3**	** 3**	3	2	2	kld26an	2	2	1	2	2	1	nlc08an	2	2	1	2	2	1	tls09an	** 2**	** 2**	** 2**	** 2**	** 2**	** 2**
eed07an	** 3**	** 3**	** 3**	** 3**	** 3**	** 3**	kmc22an	2	1	1	2	1	0	nmb28an	2	3	2	1	3	2	tpp24an	1	0	0	1	0	0
ejc1nal	** 0**	** 0**	** 0**	** 0**	** 0**	** 0**	kms29an	2	3	3	2	3	3	nmc22an	2	2	1	** 1**	** 1**	** 1**	tps16an	1	—	1	1	—	1
ejh24an	** 3**	** 3**	** 3**	3	0	1	kmw05an	** 3**	** 3**	** 3**	3	1	1	nml15an	0	1	1	0	1	1	txn1nal	1	—	0	1	—	0
emp27an	** 2**	** 2**	** 2**	** 2**	** 2**	** 2**	kps25an	0	2	1	0	2	1	nmv07an	** 3**	** 3**	** 3**	3	0	0	vaw07an	** 3**	** 3**	** 3**	** 3**	** 3**	** 3**
ess05an	1	0	0	1	0	0	ktj26an	** 3**	** 3**	** 3**	** 3**	** 3**	** 3**	oab28an	1	3	3	2	3	3	vmc1nal	1	0	0	1	0	0
eww05an	** 2**	** 2**	** 2**	** 2**	** 2**	** 2**	kxb17an	** 3**	** 3**	** 3**	** 3**	** 3**	** 3**	ovk1nal	** 0**	** 0**	** 0**	** 0**	** 0**	** 0**	wcb24an	1	0	0	1	0	0
fmb1nal	** 0**	** 0**	** 0**	** 0**	** 0**	** 0**	kxh30an	** 3**	** 3**	** 3**	** 3**	** 3**	** 3**	pat10an	2	3	3	1	3	3	wdk1nal	** 0**	** 0**	** 0**	** 0**	** 0**	** 0**
fmr17an	** 3**	** 3**	** 3**	** 3**	** 3**	** 3**	lac02an	2	1	1	2	0	1	pbd1nal	1	0	0	1	0	0	wfc07an	2	3	2	2	3	2
fxc12an	1	2	2	1	2	2	lad13an	1	2	1	1	2	1	pca1nal	1	0	0	** 0**	** 0**	** 0**	wjb06an	2	0	0	2	0	0
gdr15an	** 3**	** 3**	** 3**	3	0	0	lad1nal	** 0**	** 0**	** 0**	** 0**	** 0**	** 0**	pdo11an	2	3	3	2	3	3	wjp20an	** 3**	** 3**	** 3**	** 3**	** 3**	** 3**
gmm09an	** 3**	** 3**	** 3**	** 3**	** 3**	** 3**	lai04an	1	3	2	1	3	2	pgb16an	** 1**	** 1**	** 1**	** 1**	** 1**	** 1**	wpb30an	2	1	1	2	1	1
gms05an	2	3	3	2	3	3	lap05an	1	3	3	1	3	3	plw14an	** 2**	** 2**	** 2**	** 2**	** 2**	** 2**	wxe04an	** 3**	** 3**	** 3**	** 3**	** 3**	** 3**

## Appendix

On Table 6 perceptual assessment of GRBAS Grade and Roughness traits for 224 recordings of MEEI corpus [55] can be found.* G*1 and* R*1 are assessments of Therapist 1. The rest of evaluations are performed by Therapist 2 in two different sessions. Numbers in bold represent the* agreement subsets* while all evaluations are the* original subsets*.
